# Regulation of Immune Responses and Autoimmune Encephalomyelitis by PPARs

**DOI:** 10.1155/2010/104705

**Published:** 2010-12-22

**Authors:** Yuhong Yang, Amy E. Lovett-Racke, Michael K. Racke

**Affiliations:** ^1^Department of Neurology, Ohio State University Medical Center, Columbus, OH 43210, USA; ^2^Department of Molecular Virology, Immunology and Medical Genetics, Ohio State University Medical Center, Columbus, OH 43210, USA

## Abstract

PPARs are members of the steroid hormone nuclear receptor superfamily and play an important role in regulating inflammation as well as lipid metabolism. The PPAR subfamily has been defined as PPAR*α*, PPAR*β*/*δ*, and PPAR*γ*, each with different ligands, target genes, and biological roles. PPARs regulate the expression of target inflammatory genes through mechanisms involving both transactivation and transrepression. The anti-inflammatory properties of PPAR agonists have led to the investigation of PPAR functions in regulating autoimmune encephalomyelitis. This paper will summarize some of the general mechanisms by which PPARs regulate inflammatory gene expression and focus on the recent advances of PPAR regulation of autoimmune encephalomyelitis.

## 1. Peroxisome Proliferator-Activated Receptors (PPARs)

The nuclear receptor superfamily integrates both inflammatory and metabolic signals to maintain homeostasis in immune cells such as macrophages, dendritic cells, and lymphocytes [1, 2]. PPARs are nuclear receptors activated by fatty acids and control the expression of genes involved in both lipid metabolism and inflammation. So far, there are three isoforms that have been identified and cloned, including PPAR-*α*, PPAR-*β*/*δ*, and PPAR-*γ*, and they exhibit different tissue distribution as well as different ligand specificities. PPAR*α* was the founding member of the PPAR subfamily and was first cloned in rodents. It was shown to be activated by a diverse class of rodent hepatocarcinogens that causes proliferation of peroxisomes [3]. Subsequently, two other family members were discovered, PPAR*β*/*δ* and PPAR*γ* [4, 5]. Natural ligands for the PPARs include native and modified polyunsaturated fatty acids and eicosanoids [6–8]. Additionally, the PPARs have a large ligand-binding pocket that can accommodate a diverse range of synthetic ligands [9–11]. 

All PPARs have four main domains named A/B, C, D, and E/F. The N-terminal A/B domain has a transcriptional activating function (AF-1). The C domain, or DNA binding domain (DBD), is formed by two zinc finger-like motifs that can recognize a peroxisome proliferator response element (PPRE) on target genes. PPREs are specific DNA sequences of the repetition of a consensus hexanucleotide sequence (AGGTCA), separated by one or two nucleotides. The D domain is a hinge region that can modulate the DNA binding ability and is involved in cofactor interaction. The E/F domain is the ligand-binding domain (LBD), which is responsible for the ligand binding and is involved in the dimerization with the 9-cis retinoic acid receptor (RXR) [12].

PPARs are expressed by several different immune cells, including macrophages [13–15], T cells [16–19], B cells [20], and dendritic cells [21–23]. Other than regulation of lipid metabolism, PPARs have also been shown to play an important role in regulating immune responses and inflammation, by programming inflammatory gene expression in immune cells, including macrophages, dendritic cells, and lymphocytes [8, 24, 25]. All three members of the PPAR family have been shown to exert anti-inflammatory effects in vitro and in vivo. The anti-inflammatory effects of PPAR agonists have been observed in autoimmune diseases, including multiple sclerosis (MS) and experimental autoimmune encephalomyelitis (EAE). Although the detailed mechanisms by which PPARs regulate inflammatory responses and autoimmune encephalomyelitis are still not well established, recent studies have broadened our understanding on the transcriptional regulation of inflammatory target genes by PPARs and shed light on the mechanism of PPAR regulation of autoimmune encephalomyelitis. The positive regulation of target gene transcription by PPARs was through direct binding to the PPRE on the promoter of target genes, whereas negative regulation of target gene expression was mostly indirect, through a mechanism termed transrepression [6, 26]. This paper will summarize some general mechanisms by which PPARs regulate inflammatory gene expression and focus on the recent advances of PPAR regulation of autoimmune encephalomyelitis.

## 2. General Mechanisms of PPAR Action

### 2.1. Positive Regulation of Target Gene Expression

PPARs can both positively and negatively regulate their target gene expression. One of the mechanisms by which PPARs exert their function is through binding to a PPRE as a heterodimer with RXR in a ligand-dependent manner. Ligand-dependent activation is linked to the recruitment of coactivator complexes that modify chromatin structure and facilitate assembly of the general transcriptional machinery at the promoter [27, 28]. 

In the unliganded state, PPARs are associated with a nuclear receptor corepressor. NCoR (nuclear receptor corepressor) is among the most studied corepressors. In addition, heat shock protein-90 and the hepatitis virus B X-associated protein 2 have been shown to be associated with PPAR-*α*, which negatively regulates subsequent gene activation [29, 30]. Upon ligand activation, the PPARs undergo a conformational change that results in the dissociation from the corepressor, enabling the PPARs to bind nuclear receptor coactivators. These coactivators then act to reorganize the chromatin templates allowing the basal transcription machinery to gain access to the promoter regions and drive the transcription of target genes [24]. One example of positive regulation of inflammatory gene expression in autoimmune encephalomyelitis is the regulation of IL-4 gene by the PPAR*α* agonist gemfibrozil. Gemfibrozil induced immune deviation and protected mice from EAE. PPAR-*α* was shown to regulate the IL-4 and IL-5 genes and bind to the IL-4 promoter in the presence of steroid receptor coactivator-1 (SRC-1), suggesting transactivation of the IL-4 gene [31].

### 2.2. Negative Regulation of Target Gene Expression by Transrepression

The ligand-dependent gene repression by PPARs is mediated through an indirect regulatory mechanism, termed ligand-dependent transrepression, which is distinguished from active repression of target genes in that the repression does not depend on the binding of PPARs to PPREs in target gene promoters. The general mechanism of transrepression involves protein-protein interactions between PPARs and their target transcription factors or coregulatory complexes. Transrepression is widely used to negatively regulate gene expression but the detailed mechanism by which different nuclear receptors exert their specific transrepression varies from receptor to receptor. It is difficult to identify a unified mechanism of repression by all PPARs, since signal, cell, and/or promoter-specific mechanisms exist for all three PPAR family members. 

#### 2.2.1. Tether Transcription Factors Away from Their Binding Sites

One of the mechanisms by which PPARs transrepress their target inflammatory gene expression is to tether transcription factors away from their DNA binding sites in the target gene promoter region, which in turn inhibits gene transcription of the target inflammatory genes. This process involves physical interaction between PPARs and their target transcription factors. The inhibition of phorbol ester-induced IL-2 expression by PPAR*γ* was mediated by this mechanism. PPAR*γ* ligands, troglitazone and 15 deoxy Delta (12, 14) prostaglandin J (2) (15d-PGJ2), inhibited IL-2 production and phytohemagglutinin-inducible proliferation in human peripheral blood T cells in a dose-dependent manner. The activated PPAR*γ* physically associates with the transcription factor, nuclear factor of activated T cells (NFAT), regulating the IL-2 promoter by blocking NFAT DNA binding and transcriptional activity. The ligand-dependent binding of PPAR*γ* to NFAT correlates with the dissociation of NFAT from the IL-2 promoter [32].

#### 2.2.2. SUMOylation-Dependent Pathway

Another important transrepression pathway is the SUMOylation-dependent pathway. SUMOylation-dependent targeting of a nuclear receptor to corepressor complexes to prevent their signal-dependent clearance is likely to represent a general molecular strategy for transrepression of proinflammatory target genes [33, 34]. The SUMOylation-dependent pathway mediated transrepression of inflammatory response genes was first identified for PPAR*γ* and then extended to PPAR*α* and two additional members of the nuclear receptor family, LXR*α* and LXR*β*. In macrophages, the SUMOylation-dependent pathway was initiated by ligand-induced SUMOylation of the PPAR*γ* ligand-binding domain. This modified PPAR*γ* in turn bound to NCoR complexes associated with the promoters of target inflammatory genes, which were marked by the presence of NCoR-HDAC3-TBL corepressor complexes. These complexes prevented Ubc5 recruitment in response to lipopolysaccharide (LPS) signals, which supposedly led to the clearance of NCoR and HDAC3, and the switch from repression to transcriptional activation. As a result, NCoR complexes were not cleared from the promoter, and target genes were maintained in a repressed state [33]. 

Collectively, recent studies have defined some molecular mechanisms by which PPARs positively or negatively regulate their target inflammatory gene expression. However, the molecular mechanisms by which PPARs regulate autoimmune encephalomyelitis have not been well defined. So far, no study has been published demonstrating the detailed molecular mechanism of negative regulation of PPAR in the EAE model although Gocke et al. showed the molecular pathway of positive regulation of IL4 and IL5 gene by PPAR-*α* in EAE*.* More studies are needed in the future to elucidate the detailed mechanisms by which PPARs regulate autoimmune encephalomyelitis.

## 3. MS and EAE

**Figure 1:**
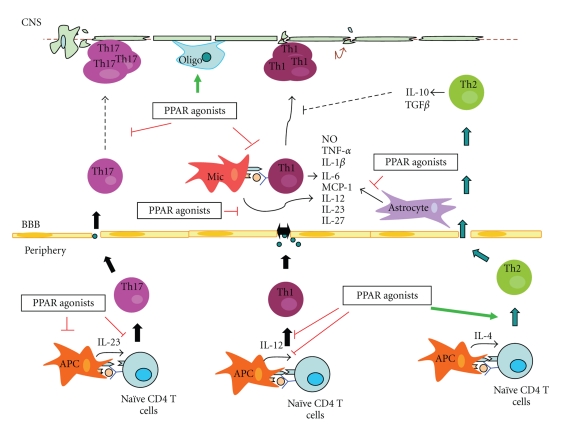
*Regulation of autoimmune encephalomyelitis by PPAR agonists. *CD4 T cells differentiate into different types of T effector cells in the periphery in response to different pathogenic microorganisms as a result of recognition of these organisms by the innate immune system. The IL-12-driven Th1 cell subset mainly produces IFN-*γ*, IL-2, and GM-CSF and plays an essential role in mediating disease. The Th2 cell subset produces IL-4, IL-5, and IL-13, which are associated with amelioration of EAE and remission in MS. The IL-23-driven Th17 cells have also been shown to be critical in the development of autoimmune diseases. PPAR agonists have been shown to suppress autoimmune encephalomyelitis by regulating the function of both immune cells and CNS-resident cells, including inhibiting Th1 and Th17 differentiation, promoting Th2 differentiation, inhibiting inflammatory cytokine production by microglia and astrocytes, and increasing the number of myelin-producing OLs. Increased expression is indicated by green arrow and decreased expression shown by red blockade.

MS is the leading cause of neurologic disability in the United States in young adults after trauma; thus, most patients suffer from the effects of MS for most of their adult life. MS is thought to be a T cell-mediated autoimmune disease of the central nervous system (CNS) with a complex genetic background. Although the precise etiology of MS is still unknown, it is generally accepted that MS begins with the formation of acute inflammatory lesions which are mediated by autoreactive T cells and B cells. The demyelinating plaques are dominated by activated T cells and macrophages associated with oligodendrocyte and myelin destruction (Figure 1).

CD4 T cells are at the center of MS pathogenesis and are the focus of MS research, given their important role in mediating disease. CD4 T cells differentiate into different types of T effector cells in the periphery in response to different pathogenic microorganisms as a result of recognition of these organisms by the innate immune system. It has been suggested for more than two decades that there are two different types of CD4 T helper cells, Th1 and Th2 cells. The Th1 cell subset mainly produces IFN-*γ*, IL-2 and GM-CSF, while the Th2 cell subset produces IL-4, IL-5 and IL-13 [35–38]. Th1 cells have been implicated in a variety of autoimmune diseases, including MS [39–41]. Conversely, Th2 cells control infections by extracellular microbes, and cytokines produced by Th2 cells mediate helper T cell functions for antibody production and mediate the immunopathology of allergic responses. Early studies suggested that the IFN-*γ* producing Th1 CD4 T cells, which were driven by IL-12, played an essential role in mediating disease, while Th2 cytokines such as IL-4 were associated with amelioration of EAE and remission in MS. More recently, Th17 cells have been identified as a new CD4 T cell lineage. In vivo, Th17 cells were driven by IL-23 although, in vitro, they were induced by TGF-*β* and IL-6. Th17 cells also have been shown to be critical in the development of autoimmune diseases. Studies have been done to define the roles of different T cell subpopulations in MS pathogenesis and focus on how to manipulate pathogenic Th1 and Th17 cells and related cytokines to suppress disease. 

EAE is an inflammatory demyelinating disease mediated by myelin-specific Th1 and Th17 CD4 lymphocytes. EAE is characterized by relapsing paralysis, CNS inflammation, and demyelination. It has been used as a model for MS for several decades, since it shares clinical and immunopathological similarities to MS. EAE can be induced in mice by immunization with various myelin proteins or peptides emulsified in CFA or by the transfer of activated myelin-specific CD4 Th1 lymphocytes into naive recipients. 

The anti-inflammatory properties of PPAR agonists have led to the investigation of PPAR functions in regulating autoimmune encephalomyelitis, hoping to develop new therapeutic strategies for MS. Many studies have been performed to test the effects of different PPAR agonists in regulating EAE and have shown very promising results. Some of the targets of PPARs in MS pathogenesis are shown in Figure 1. Based on these promising animal data, a PPAR agonist has been tested in a clinical trial as an adjunctive treatment for MS patients. In the next three sections, we are going to discuss the regulation of autoimmune encephalomyelitis by PPAR*γ*, PPAR*α*, and PPAR*β*/*δ*, respectively.

## 4. Regulation of Autoimmune Encephalomyelitis by PPAR*γ*

### 4.1. PPAR*γ* Agonists Suppress EAE

Several PPAR-*γ* agonists have been shown to ameliorate EAE. Troglitazone was shown to ameliorate MOG 35-55-induced EAE in C57BL/6 mice, and troglitazone treatment during the effector phase is more effective than when it is given during the induction phase [42]. Administration of another PPAR*γ* agonist, 15d-PGJ2, before and at the onset of clinical signs of EAE significantly reduced the severity of disease in B10 PL mice. More importantly, culture of encephalitogenic T cells with 15d-PGJ2 reduced their ability to adoptively transfer EAE, suggesting that PPAR-*γ* ligands may regulate T cell encephalitogenicity in vitro [43]. Furthermore, the combination of 15d-PGJ2 and 9-cis-retinoic acid (RA), the ligand for RXR, resulted in enhanced amelioration of disease, suggesting that combination of RXR-specific ligands and PPAR*γ* ligands may be highly effective in the treatment of autoimmune demyelinating diseases such as MS [44]. Similarly, in SJL/J mice, 15d-PGJ2 or Ciglitazone decreased the duration and clinical severity of active immunization and adoptive transfer models of EAE [45]. Orally administered pioglitazone was also shown to reduce the incidence and severity of monophasic, chronic disease in C57BL/6 mice and of relapsing disease in B10.PL mice. Pioglitazone also reduced clinical signs when it was provided after disease onset. The suppression of clinical signs was paralleled by decreased lymphocyte infiltration, lessened demyelination, reduced chemokine and cytokine expression, and increased inhibitor of *κ*B (I*κ*B) expression in the brain [46]. Another PPAR*γ* aonist, rosiglitazone, when used to treat DCs, was able to prevent EAE development in mice [47]. 

On the other hand, PPAR*γ* antagonists exacerbated EAE. Treatment with PPAR*γ* antagonists, Bisphenol A diglycidyl ether (BADGE), or 2-Chloro-5-nitro-N-(4-pyridyl)benzamide (T0070907) increased the severity and duration of EAE in C57BL/6 wild-type and PPAR*γ*-deficient mice. The exacerbation of EAE was associated with an augmented neural antigen-induced T cell proliferation, IFN*γ* production, and Th1 differentiation [48]. Furthermore, BADGE and benzamide (T0070907) reversed the inhibition of EAE by the PPAR*γ* agonists, Ciglitazone and 15d-PGJ2, in C57BL/6 wild-type and PPAR*γ*+/− mice. The reversal of EAE was associated with restoration of neural antigen-induced T cell proliferation, IFN*γ* production, and Th1 differentiation inhibited by Ciglitazone and 15d-PGJ2 [49]. 

Together, these data demonstrated that PPAR*γ* played an important role in regulating autoimmune encephalomyelitis in vivo and suggested that PPAR*γ* agonists might be a new therapeutic treatment for autoimmune demyelinating diseases such as MS.

### 4.2. EAE in PPAR*γ*-Deficient Mice

It is controversial whether PPAR*γ* agonists such as 15d-PGJ2 require PPAR*γ* for their anti-inflammatory function, because there are studies showing PPAR*γ*-independent mechanisms in the induction of anti-inflammatory effects by 15d-PGJ2 [50]. PPAR*γ*-deficient heterozygous mice were used to demonstrate the function of endogenous PPAR*γ* in EAE. In the endogenous state, the PPARs are likely occupied by their fatty acid ligands, which may be produced at sites of inflammation. PPAR*γ*-deficient heterozygous mice developed an exacerbated course of EAE with prolonged clinical signs compared to wild-type littermates. The exacerbation was associated with an increased expansion of CD4 and CD8 T cells and expression of CD40 and MHC class II molecules in response to antigen, confirming PPAR*γ* as a critical regulator of EAE and perhaps MS [51].

### 4.3. Role of PPAR*γ* in Regulating Immune Cells in EAE

Since studies in vivo showed PPAR*γ* agonists inhibited CNS inflammation and demyelination in EAE, studies were done to elucidate the potential therapeutic mechanisms. Troglitazone has been shown to attenuate the inflammation and decreased the clinical signs through the attenuation of proinflammatory cytokine gene expression in the spinal cord [42]. In addition to this, additional studies have been published to show the different effects of PPAR*γ* agonists on immune cells and CNS resident cells in EAE as described below. 

#### 4.3.1. Antigen-Presenting Cells

PPAR*γ* agonists were shown to regulate the function of antigen-presenting cells, including monocyte/macrophages and dendritic cells. 15d-PGJ2 was shown to inhibit phorbol ester-induced nitric oxide (NO), TNF-*α*, IL-1, and IL-6 production by cells of the monocyte/macrophage lineage, in part by antagonizing the activities of transcription factors such as AP-1 and NF-*κ*B [14, 15]. Another PPAR*γ* agonist, rosiglitazone, was shown to interfere with NF-*κ*B activation in murine DCs. As a result, treated DCs showed impaired maturation and a reduced capacity to activate antigen-specific T cells and were able to prevent EAE development in mice [47].

#### 4.3.2. T Cells

Since MS and EAE are suspected T cell-mediated autoimmune diseases, studies have been conducted to determine how PPARs regulate T cell function. 15d-PGJ2 was shown to inhibit the proliferation of Ag-specific T cells from myelin basic protein Ac1-11 TCR-transgenic mice and suppress IFN-*γ*, IL-10, and IL-4 production by lymphocytes. Similarly, the disease inhibition with 15d-PGJ2 or Ciglitazone in SJL/J mice was associated with a decrease in IL-12 production and differentiation of neural antigen-specific Th1 cells. Treatment of activated T cells with PPAR*γ* agonists in vitro inhibited IL-12-induced activation of the JAK-STAT signaling pathway and Th1 differentiation [45]. Orally administered pioglitazone was also shown to reduce the antigen-dependent IFN-*γ* production from EAE-derived T cells [46]. 

In addition to pathogenic Th1 cells, Th17 cells have also been shown to be pathogenic in MS and EAE. One recent study identified PPAR*γ* as a key negative regulator of human and mouse Th17 differentiation. PPAR*γ* activation in CD4 T cells selectively suppressed Th17 differentiation through inhibition of TGF-*β*/IL-6 induced ROR*γ*t expression, but not differentiation into Th1, Th2, or regulatory T cells. More importantly, human CD4 T cells from healthy controls and MS patients were strongly susceptible to PPAR*γ*-mediated suppression of Th17 differentiation, suggesting that PPAR*γ* is a promising molecular target for specific immunointervention in Th17-mediated autoimmune diseases such as MS [52].

### 4.4. Role of PPAR*γ* in CNS-Resident Cells in EAE

Other than regulating immune responses, PPAR*γ* agonists were also shown to regulate the functions of CNS-resident cells, including microglia and astrocytes. It has been shown that PPAR*γ* agonists modulate EAE, at least in part, by inhibiting the activation and cytokine production of microglia and astrocytes. 15d-PGJ2 together with 9-cis retinoic acid potently inhibited microglial cell activation and inhibit EAE development in mice [53]. Three TZDs, rosiglitazone, pioglitazone, ciglitazone, and 15d-PGJ2, were all effective in inhibiting production of NO, the proinflammatory cytokines TNF-*α*, IL-1*β*, and IL-6, and the chemokine MCP-1 from microglia and astrocytes [54, 55]. 15d-PGJ2 and rosiglitazone inhibited the induction of IL-12p40, IL-12p70 (p35/p40), IL-23 (p19/p40), and IL-27p28 proteins by LPS-stimulated primary microglia. 15d-PGJ2 potently suppressed IL-12p40, IL-23, and IL-27p28 production by primary astrocytes, whereas rosiglitazone suppressed IL-23 and IL-27p28, but not IL-12p40 in these cells [56]. These effects on CNS-resident cells might contribute to the suppression of EAE by PPAR*γ* agonists.

### 4.5. PPAR*γ* Agonists and MS

PPAR*γ* agonist pioglitazone was tested as an add-on therapy with interferon-*β* in a small cohort of relapsing remitting MS (RRMS) patients. RRMS patients taking IFN*β*-1*α* were randomized to treatment with pioglitazone (30 mg daily, p.o.) or placebo and monitored clinically by EDSS and by MRI for 1 year. After 1 year, there were no significant differences in clinical signs as assessed by EDSS; however, MRI showed a significant reduction in gray matter atrophy and a trend for reduced lesion burden in the treatment group. These data suggested some beneficial effects for RRMS patients, and further trials need to be performed to establish clinical efficacy [57].

PPAR*γ* involvement in autoimmune encephalomyelitis was also implicated by a population-based study in MS patients. The Ala allele of the PPAR*γ* Pro12Ala polymorphism was strongly associated with delayed disease onset (44.1 ± 5.3 years versus 34.5 ± 4.2 years; *P* = .006). This study demonstrated that the Pro12Ala polymorphism resulting in an amino acid exchange in the N-terminal sequence of PPAR*γ* may influence the onset of MS [58].

In summary, data from EAE studies showed that PPAR*γ* agonists were able to suppress disease severity by regulating the functions of both immune cells and CNS-resident cells (Table 1), supporting PPAR*γ* agonists as an effective treatment of autoimmune demyelinating diseases such as MS. Small-scale clinical data further confirmed that the PPAR*γ* agonist pioglitazone maybe beneficial for RRMS patients. More clinical studies are needed to further establish clinical efficacy.

## 5. Regulation of Autoimmune Encephalomyelitis by PPAR*α*

PPAR*α* is expressed in different immune cells, including monocytes/macrophages, T cells, and B cells and plays an important role in regulating inflammation and cytokine production. PPAR*α* agonists have also been tested in the treatment of EAE and shown to be protective [24]. However, recent studies showed, other than sharing the common anti-inflammatory effects of all three PPAR subtypes, PPAR*α* had specific effects in inducing immune deviation in EAE. This specific regulation of immune deviation made these PPAR*α* agonists very attractive candidates to be used therapeutically in treating Th1-mediated autoimmune diseases, including MS, in addition to their excellent track history as oral agents used to treat hypertriglyceridemia. Here we are going to focus on several recent studies demonstrating PPAR*α* regulation of immune deviation, gender differences, and its role in CNS-resident cells (Table 2). 

### 5.1. Immune Deviation Induced by PPAR*α* Agonists

#### 5.1.1. Immune Deviation

Th1 and Th2 cells are two distinct CD4 T cell lineages, and they play different roles in autoimmune diseases, including MS and EAE. Autoimmune diseases can be divided into those mediated by Thl cells with primarily inflammatory manifestations and those mediated by Th2 cells whose manifestations are secondary to autoantibody containing immune complexes [66]. Immune deviation was a term used to characterize an immune response where Th2 cells predominate, and one approach to the immunotherapy of inflammatory autoimmune disease, including MS, was the antigen-specific deviation of an immune response dominated by a Th1 response to a Th2 response.

#### 5.1.2. PPAR*α* Agonists Ameliorate EAE by Inducing Immune Deviation

The PPAR*α* agonist gemfibrozil was shown to regulate immune responses by promoting the deviation of immune responses dominated by a pathogenic Th1 response to a nonpathogenic Th2 response [31, 60, 67]. Lovett-Racke et al. demonstrated that PPAR*α* agonists induced a shift in cytokine production from Th1 cytokines to Th2 cytokines in both mouse and human T cells and protected mice from EAE. PPAR*α* agonists increased the production of the Th2 cytokine, IL-4, and suppressed proliferation by TCR transgenic T cells specific for the myelin basic protein Ac1-11 peptide. Oral administration of PPAR*α* agonists gemfibrozil and fenofibrate inhibited the clinical signs of EAE. More importantly, the PPAR*α* agonist gemfibrozil shifted the cytokine secretion of human T cell lines from IFN*γ* secretion to IL-4 secretion [59]. Gocke et al. studied the molecular mechanisms by which PPAR*α* agonists induce immune deviation and protect mice from EAE. They demonstrated that PPAR*α* agonists directly activated Th2 cytokine IL-4 gene expression by directly binding to the IL-4 promoter region. Gemfibrozil treatment increased Th2 transcription factor GATA-3 expression and decreased Th1 transcription factor T-bet expression in vitro and directly ex vivo. For the first time, they showed that PPAR*α* regulated the IL-4 and IL-5 genes and bound the IL-4 promoter in the presence of the steroid receptor coactivator-1 [31].

#### 5.1.3. Is the Effect Receptor Dependent or Not?

Gocke et al. showed that the protective effects of PPAR*α* agonists in EAE occurred in a receptor-dependent manner [31]. Dasgupta et al. observed similar effects with the PPAR*α* agonist Gemfibrozil in mice, including switching of a Th1 profile to a Th2 profile, inhibiting T-bet expression, stimulating GATA3 expression, and inhibiting the encephalitogenicity of antigen-primed T cells. However, they suggested the switch of immune response from a Th1 to a Th2 profile by PPAR*α* agonists was receptor independent, as the drug was equally effective in treating EAE in PPAR*α*-deficient and well as wild-type mice [60]. Similarly, Cunard et al. showed that treatment with WY14,643 and other fibrates led to marked increases in supernatant concentrations of IL-4. They also showed that this effect on IL4 production was largely through a PPAR*α*-independent mechanism, since WY14,643 induced IL-4 expression in splenocytes from PPAR*α*-deficient mice [19]. Further studies are needed to elucidate the receptor dependency of PPAR*α* agonists.

### 5.2. PPAR*α* and Gender Differences

PPAR*α* could be one of the genes mediating gender differences in EAE. PPAR*α* expression in T cells is higher in male mice compared to female mice, and this expression is reduced by castration and increased by *α*-DHT treatment. The deficiency of PPAR*α* gene expression resulted in higher IFN-*γ* and TNF*α* production by T cells in male mice. Male but not female PPAR*α*-deficient mice developed more severe EAE that was restricted to the acute phase of disease. These findings provide a molecular basis for why males may be less prone to developing Th1-mediated autoimmunity [67].

Women are more susceptible than men to develop autoimmune diseases, including MS. In MS, twice as many women as men develop the disease. This may be related to the fact that women have more robust immune responses than men although the exact mechanism is not understood [68]. A study showed women to be more prone than men to develop Th1-polarized responses directed against myelin antigens during MS [69]. Whether PPAR*α* is responsible for the gender differences in MS susceptibility remains to be determined.

### 5.3. PPAR*α* Regulation of CNS-Resident Cells in EAE

Moreover, PPAR*α* agonists were also shown to regulate CNS-resident cells and the protective effects of PPAR*α* agonists in EAE were in part through effects on CNS cells. Microglia cells are resident CNS cells that may serve as antigen-presenting cells. Activated microglia exhibit increased pathogenic cytokine production and increased synthesis of NO, which may contribute to axonal degradation in MS. Xu et al. showed that PPAR*α* agonists inhibited microglia production of NO, IL-1*β*, and TNF-*α*, which were potentially toxic to cells including myelin-producing oligodendrocytes. In addition, these agonists inhibit microglial production of Th1- and Th17-promoting cytokines, IL-12 and IL-23 [61]. PPAR*α* agonists also suppressed microglia production of MCP-1, a chemokine that plays an important role in modulating monocyte infiltration into the CNS in MS [70]. Similar effects were observed in LPS-stimulated astrocytes. A combination of 9-cis RA and the PPAR*α* agonists fenofibrate or gemfibrozil cooperatively inhibited NO, TNF-*α*, IL-1*β*, IL-6, and MCP-1 production by these cells [62]. Thus, PPAR*α* agonists could also modify cytokine expression in the CNS during inflammation such as that observed in EAE or MS.

## 6. Regulation of Autoimmune Encephalomyelitis by PPAR*β*/*δ*

PPAR*β*/*δ* is the predominant PPAR isotype in brain. However, the exact functions of PPAR*β*/*δ* are not yet well understood, but it is likely to play a role in cell proliferation [71], differentiation, survival, lipid metabolism, and development [72, 73]. 

### 6.1. PPAR-*β*/*δ*-Specific Agonists and EAE

A protective effect in EAE was reported for a PPAR-*β*/*δ*-specific agonist, and this protection was suggested to be due to a reduction in glial inflammation. Polak et al. showed that oral administration of the selective PPAR*δ* agonist GW0742 reduced clinical signs in actively immunized C57BL/6 mice, especially when it was administered during disease progression [63]. The protective effect of GW0742 was receptor dependent, since no amelioration of EAE clinical scores was observed in PPAR*δ*-deficient mice [64]. RT-PCR analysis showed that GW0742 increased expression of some myelin genes. GW0742 reduced astroglial and microglial inflammatory activation and IL-1*β* levels in EAE brain. 

Other than GW0742, two other PPAR*β*/*δ* agonists, GW501516 and L165041 were shown to ameliorate MOGp35-55-induced EAE in C57BL/6 mice by blocking IFN-*γ* and IL-17 production by Th1 and Th17 cells [65]. GW 501516 was also tested for its capacity to protect from antibody-mediated demyelination. However, GW 501516 did not protect against antibody-mediated demyelination although it showed some anti-inflammatory activity [74]. 

The regulation of EAE by PPAR*β* was further confirmed by a study in Steroid receptor coactivator-3-(SRC-3) deficient mice. SRC-3 is a member of the p160 family of coactivators that interact with nuclear receptors to enhance their transactivation in a ligand-dependent manner. SRC-3 deficiency significantly inhibited the disease severity of EAE. However, these effects are not caused by inhibition of peripheral T cell response, but by upregulation of PPAR*β* in the CNS, which induced an alternative activation state of microglia in SRC-3 deficient mice. These alternatively activated microglia inhibited CNS inflammation through inhibition of proinflammatory cytokines and chemokines, such as TNF-*α*, IFN-*γ*, CCL2, CCL3, CCL5, and CXCL10 as well as upregulation of the anti-inflammatory cytokine IL-10 and opsonins [75].

### 6.2. PPAR*β*/*δ* Regulation of Immune Responses

Treatment of T-cells with GW0742 either in vivo or in vitro did not reduce Th1 cytokine IFN*γ* production [63]. However, a study showed that the PPAR*δ* agonists, GW501516 and L165041, ameliorated EAE by blocking IFN*γ* and IL-17 production by Th1 cells and Th17 cells and was associated with a decrease in IL-12 and IL-23 and an increase in IL-4 and IL-10 expression in the CNS and lymphoid organs [65]. 

The PPAR*β*/*δ* regulation of immune cells was further confirmed by one recent study in PPAR*β*/*δ*-deficient mice. PPAR-*β*/*δ*-deficient mice developed a severe inflammatory response during EAE characterized by a striking accumulation of IFN-*γ*+ IL17A- and IFN-*γ*+ IL-17A+ CD4+ cells in the spinal cord, which resulted from immune system aberrations including enhanced Th cell expansion, cytokine production, and T-bet expression and enhanced expression of IL-12 family cytokines by myeloid cells. This data strongly suggests that PPAR-*δ* serves as an important molecular brake for the control of autoimmune inflammation [76] (Table 3).

### 6.3. PPAR*β*/*δ* and Oligodendrocyte (OL) Maturation

The special feature of PPAR*δ* function is that PPAR*δ* agonists are more effective when administered during later stages of disease and they increase myelin gene expression [63], which suggested that they might affect OL maturation. Vittoria Simonini et al. demonstrated that PPAR*δ* played a role in OPC maturation. GW0742 was shown to increase the number of myelin-producing OLs in OPCs, and this was receptor dependent, since OLs were reduced in PPAR-deficient OPCs [64].

## 7. Conclusion

PPARs are lipid-activated transcription factors that have emerged as key regulators of both lipid metabolism and inflammation. They exert positive and negative controls over the expression of a range of inflammatory genes. The anti-inflammatory properties of PPARs make them attractive targets for intervention in human autoimmune diseases, including MS. A growing body of literature suggested that PPAR agonists could be used therapeutically in autoimmune diseases such as MS as a preliminary clinical study has suggested. Further studies will be required to fully understand the complicated mechanisms of PPAR regulation of immune responses and autoimmune encephalomyelitis.

## Figures and Tables

**Table 1:** The effects of PPAR*γ* agonists in EAE.

Agonists (concentration)	Effects on immune cells	Effects on CNS cells	References
15d-PGJ2 (100 *υ*g/kg/day to 1 mg/kg/day)	Suppresses T cell proliferation Suppresses IFN-*γ*, IL12 and IL4 production. Inhibits Th1 differentiation.	Inhibits CD40 expression on microglial cells. Inhibits production of NO, TNF-*α*, IL-1*β*, and IL-6, and MCP-1, and IL12, IL23, IL27 expression from microglia and astrocytes.	Diab et al. [43, 44], Natarajan and Bright [45], Drew et al. [53], Storer et al. [54, 55], Xu and Drew [56]

Pioglitazone (5–10 mg/kg/day)	Reduces T-cell activation. Suppresses Th17 differentiation in CD4 cells. Inhibit ROR*γ*t expression in T cells.	Inhibits production of NO, TNF-*α*, IL-1*β*, and IL-6, and MCP-1 from microglia and astrocytes.	Feinstein et al. [46], Klotz et al. [52], Drew et al. [53], Storer et al. [54, 55]

Rosiglitazone (5–10 mg/kg/day)	Inhibits NF-Kb Activation in DC.	Inhibits production of NO, TNF-*α*, IL-1*β*, and IL-6, and MCP-1 from microglia and astrocytes. Inhibits IL12, IL23 and IL27 expression in microglia. Inhibits IL23 and IL27 expression in astrocytes.	Iruretagoyena et al. [47], Feinstein et al. [46], Drew et al. [53], Storer et al. [54, 55], Xu and Drew [56]

Ciglitazone (50–100 ug/kg/day)	Inhibit IL-12 production in macrophages and Th1 differentiation.	Inhibit IL-12 production in microglial cells. Inhibits production of NO, TNF-*α*, IL-1*β*, and IL-6, and MCP-1 from microglia and astrocytes.	Natarajan and Bright [45], Drew et al. [53], Storer et al. [54, 55]

Troglitazone (50–100 mg/kg/day)		Suppresses TNF-*α* expression in spinal cord.	Niino et al. [42]

**Table 2:** The effects of PPAR*α* agonists in EAE.

Agonists (concentration)	Effects on immune cells	Effects on CNS cells	References
Gemfibrozil (500 ug/day)	Suppress lymphocyte proliferation. Increase IL4 production in T cells. Inhibit IFN*γ* production. Inhibit the encephalitogenicity of MBP-primed T cells. Switch the immune response from a Th1 to a Th2 profile. Increase GATA-3 expression and decrease T-bet expression. Regulate the IL-4 and IL-5 genes.	Reduce NO production by microglia. Inhibit IL-1*β* and IL-6 production by astrocytes.	Lovett-Racke [59], Dasgupta et al. [60], Gocke et al. [31]

Fenofibrate	Suppress lymphocyte proliferation	Inhibit NO production by microglial cells and astrocytes. Inhibit TNF-*α* expression, IL-1*β* and IL-6 production by astrocytes. Inhibit NF-*κ*B binding activity in astrocytes. Repress IL-12p40, IL-12p70, IL-23 and IL-27p28 production by microglia.	Lovett-Racke [59], Xu et al. [61, 62]

Ciprofibrate	Suppress lymphocyte proliferation. Increase IL4 production in T cells.	Inhibit IL-1*β* and IL-6 production by astrocytes	Lovett-Racke [59], Xu et al. [62]

WY 14643	Increase IL4 production in splenocytes	Inhibit NO production by astrocytes. Inhibit TNF-*α* expression, IL-1*β* and IL-6 production by astrocytes.	Xu et al. [62], Cunard et al. [18, 19]

**Table 3:** The effects of PPAR*β*/*δ* agonists in EAE.

Agonists (Concentration)	Effects on immune cells	Effects on CNS cells	References
GW0742 (10 mg/kg/day)		Reduce astroglial and microglial inflammatory activation and IL-1*β* level in brain. Increase the number of myelin-producing OLs. Increase noggin protein expression in both OPCs and enriched astrocyte cultures.	Polak et al. [63], Simonini et al. [64]

GW501516 (25–100 ug/day)	Inhibit the expression of IFN-*γ*, IL-17, T-bet, IL-12 and IL-23. Increase the expression of IL-4 and IL-10.		Kanakasabai et al. [65]

L165041 (25–100 ug/day)	Inhibit the expression of IFN-*γ*, IL-17, T-bet, IL-12 and IL-23. Increase the expression of IL-4 and IL-10.		Kanakasabai et al. [65]
